# Integrated mRNA and microRNA analysis identifies genes and small miRNA molecules associated with transcriptional and post-transcriptional-level responses to both drought stress and re-watering treatment in tobacco

**DOI:** 10.1186/s12864-016-3372-0

**Published:** 2017-01-10

**Authors:** Qiansi Chen, Meng Li, Zhongchun Zhang, Weiwei Tie, Xia Chen, Lifeng Jin, Niu Zhai, Qingxia Zheng, Jianfeng Zhang, Ran Wang, Guoyun Xu, Hui Zhang, Pingping Liu, Huina Zhou

**Affiliations:** 1 Zhengzhou Tobacco Research Institute, Zhengzhou, 450001 China; 2 Key Laboratory of Cultivation and Protection for Non-wood Forest Trees, Ministry of Education, Central South University of Forestry and Technology, Changsha, 410000 China; 3 College of Life Science and Technology, Central South University of Forestry and Technology, Changsha, 410000 China; 4 School of Life Sciences, Central China Normal University, Wuhan, 430079 China; 5 Key Laboratory of Biology and Genetic Resources of Tropical Crops, Institute of Tropical Bioscience and Biotechnology, Chinese Academy of Tropical Agricultural Sciences, Haikou, 571101 China

## Abstract

**Background:**

Drought stress is one of the most severe problem limited agricultural productivity worldwide. It has been reported that plants response to drought-stress by sophisticated mechanisms at both transcriptional and post-transcriptional levels. However, the precise molecular mechanisms governing the responses of tobacco leaves to drought stress and water status are not well understood. To identify genes and miRNAs involved in drought-stress responses in tobacco, we performed both mRNA and small RNA sequencing on tobacco leaf samples from the following three treatments: untreated-control (CL), drought stress (DL), and re-watering (WL).

**Results:**

In total, we identified 798 differentially expressed genes (DEGs) between the DL and CL (DL vs. CL) treatments and identified 571 DEGs between the WL and DL (WL vs. DL) treatments. Further analysis revealed 443 overlapping DEGs between the DL vs. CL and WL vs. DL comparisons, and, strikingly, all of these genes exhibited opposing expression trends between these two comparisons, strongly suggesting that these overlapping DEGs are somehow involved in the responses of tobacco leaves to drought stress. Functional annotation analysis showed significant up-regulation of genes annotated to be involved in responses to stimulus and stress, (e.g., late embryogenesis abundant proteins and heat-shock proteins) antioxidant defense (e.g., peroxidases and glutathione S-transferases), down regulation of genes related to the cell cycle pathway, and photosynthesis processes. We also found 69 and 56 transcription factors (TFs) among the DEGs in, respectively, the DL vs. CL and the WL vs. DL comparisons. In addition, small RNA sequencing revealed 63 known microRNAs (miRNA) from 32 families and 368 novel miRNA candidates in tobacco. We also found that five known miRNA families (miR398, miR390, miR162, miR166, and miR168) showed differential regulation under drought conditions. Analysis to identify negative correlations between the differentially expressed miRNAs (DEMs) and DEGs revealed 92 mRNA-miRNA interactions between CL and DL plants, and 32 mRNA-miRNA interactions between DL and WL plants.

**Conclusions:**

This study provides a global view of the transcriptional and the post-transcriptional responses of tobacco under drought stress and re-watering conditions. Our results establish an empirical foundation that should prove valuable for further investigations into the molecular mechanisms through which tobacco, and plants more generally, respond to drought stress at multiple molecular genetic levels.

**Electronic supplementary material:**

The online version of this article (doi:10.1186/s12864-016-3372-0) contains supplementary material, which is available to authorized users.

## Background

Drought stress is one of the most severe environmental problems that significantly threaten agriculture. It limits the growth, development, and ultimately the yields of crop plants worldwide [1]. The mechanisms of plants to response and adapt the water deficient condition at both cellular and molecular levels include the increasing of stomatal resistance, the developing of deeper root system to obtain more water and the activating other stress-response mechanisms to re-establish cellular homeostasis and protect cellular machinery from the oxidative stresses imposed by prolonged drought stress [2–5].

A series of complicated molecular mechanisms are known to be involved in drought-stress responses in plants. The best-known example of this is the signaling associated with abscisic acid (ABA). Endogenous ABA levels were increased and ABA-dependent and ABA-independent transcriptional regulatory networks were induced under drought stress conditions [6]. In addition, a class of short endogenous non-coding RNAs termed miRNAs also involved in the plant biological processes to regulate gene expression at the post-transcriptional level under drought-stress condition [7]. For example, ABA treatment and drought stress induces the accumulation of miR159, and this this miRNA molecule targets MYB transcription factors (TFs) that positively regulate ABA responses during seed germination in *Arabidopsis*. miR159 is part of a negative feedback loop that regulates ABA responses [8]. Li et al. found that miR169a and miR169c are substantially down-regulated by drought and noted that this leads to enhanced drought tolerance in *Arabidopsis* by increasing the expression of *NFYA5* (a target of miR169), which is a crucially-important transcription factor that regulates the expression of a number of drought-responsive genes [9]. Studies have also found that miRNA169 plays important roles in drought responses in rice and tomato [10, 11].

Recent advances in sequencing technology have facilitated the discovery of new drought-response genes and small RNAs in plants. Transcriptome sequencing (mRNA-Seq) approaches have been successfully applied to study gene expression patterns under drought stress conditions in various plants, including *Arabidopsis* [12], potato [13], rice [14], soybean [15], maize [16], *Cynanchum komarovii* [17], *Citrullus colocynthis* [18], and *Brassica napus* [19]. Moreover, novel drought-stress-related miRNAs have been identified with small RNA sequencing technology in rice [20], wheat [21], sugarcane [22], *Medicago truncatula* [23], and potato [24].

Tobacco is an economically-important crop grown in many regions around the world. The draft sequences of the genomes of two tobacco species, *Nicotiana tabacum* [25] and *Nicotiana benthamiana* [26], provide a framework for the identification and functional characterization of genes and genetic networks in tobacco to enable crop improvement and basic research. Improving tolerance to drought stress in tobacco and other crops is of great economic importance. Increased understanding of the biochemical and molecular basis of plant drought-stress response processes, including studies performed at a whole-genome level should help identify integrated biological pathways involved drought responses, and such knowledge holds great promise for improving crop yields. Although several studies have documented gene expression or miRNA profiling under drought-stress treatment of tobacco [27–29], our focus here is the integrated analysis of both mRNA and miRNA profiling in tobacco under both drought stress and re-watering treatments, which should allow a high-resolution picture of the interactions that occur between transcriptional and post-transcriptional regulation during plant drought-stress responses.

To better understand the molecular basis of drought-stress responses in tobacco, we analyzed sequence data from both mRNA and small RNA libraries prepared from leaves of tobacco (*Nicotiana tabacum*) from three treatments: untreated-control condition (CL), drought stress (DL), and re-watering treatment (WL). These sample libraries were sequenced with the Illumina Hiseq platform. We compared the gene expression and miRNA profiles of tobacco leaves subjected to drought stress and re-watering treatment. The integrated analysis of mRNA and small RNA in our study provides a view of candidate drought-responsive genes and miRNA molecules in tobacco, and these can potentially be used in marker-assisted selection and in the development of drought-tolerant tobacco lines.

## Results

### Gross phenotype and physiological analyses of tobacco plants

**Fig. 1:**
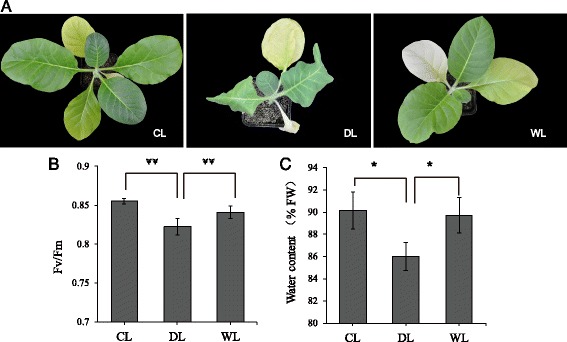
Gross phenotypes and physiological analyses of tobacco plants from the control, drought stress, and re-watering treatment groups. The growth status of control, drought-stress, and re-watering plants (**a**). Bar graph depicting the results from a chlorophyll fluorescence measurement of CL, DL, and WL leaves (**b**). Bar graph depicting the water content measurement results for CL, DL, and WL leaves (**c**). Data are presented as means ± S.D. (*n* = 7). Student’s t-test,** *P*-value < 0.01 considered highly significant; * *P*-value < 0.05 considered significant

For these experiments, plants of the three treatment groups (CL, DL, and WL) were grown in pots in a greenhouse, and the treatment period lasted for 10 days (sample collection on 10 day). CL plants were watered with 1000 mL water on days 1, 4, and 7. DL plants received no water for ten days. WL plants were re-watered, to full soil media saturation, on day 7. Note that the WL group plants were exhibiting severe wilting of leaves by day 7; the period from the re-watering on day 7 to sample collection on day 10 was represented the ‘re-watering recovery phase’ from drought. Figure 1a presents photographs of plants from the CL, DL, and WL groups at on day 10. The CL plants grew regularly and their leaves remained green. The leaves of the DL plants were turned yellow and wilted. WL plants were able to recover from wilting.

We conducted chlorophyll fluorescence and water content measurement in order to profile the physiological status of CL, DL, and WL tobacco leaves (see “Methods” section for experimental details). The result showed that the potential efficiency of PSII photochemistry (Fv/Fm) for attached DL leaves was significantly (*p* < 0.01) lower than that of CL or WL leaves (Fig. 1b) through the chlorophyll fluorescence analysis. The water content analysis indicated that leaves of the DL condition had significantly (*p* < 0.05) less water than did the CL or WL leaves (Fig. 1c). These phenotypic observations and physiological measurements clearly demonstrate that the DL plants were experiencing drought conditions and were in a highly drought-stressed state. The WL plants clearly underwent some degree of recovery from drought stress prior to sampling on day 10.

### mRNA expression profiling in tobacco leaves

In order to profile the expression of genes in tobacco leaves in response to drought stress, total RNA was extracted from leaves of plants grown in the following conditions: control (CL), drought (DL), and re-watering (WL). Poly-A enriched fractions (mRNA) were used to construct libraries for Illumina sequencing (See Methods section for details). There were a total of 35,609,580, 19,971,784, and 18,429,743 paired-end reads in the three libraries (CL, DL, and WL), of which 34,185,801 (96.01% of clean reads), 19,061,203 (95.44%), and 17,550,820 (95.23%), were mappable and could be aligned to reference genome (Table 1). On average, 85.38% of the clean reads could be mapped to annotated genes.

**Table 1:** Summary statistics of the mRNA and small RNA sequencing results for the three tobacco leaf sample libraries

	Category	CL	DL	WL
mRNA-Seq	Clean reads	35,609,580	19,971,784	18,429,743
	Reads mapped to Genome	34,185,801 (96.01%)	19,061,203 (95.44%)	17,550,820 (95.23%)
	Reads mapped to transcripts	30,885,615 (86.73%)	16,674,395 (83.49%)	15,833,985 (85.92%)
sRNA-Seq	Clean sRNA reads	30,374,218	24,325,393	23,388,818
	Reads mapped to Genome	30,055,199 (98.95%)	23,907,462 (98.28%)	23,094,708 (98.74%)
	Known miRNAs	49	44	50
	Novel miRNA	175	174	192

**Fig. 2:**
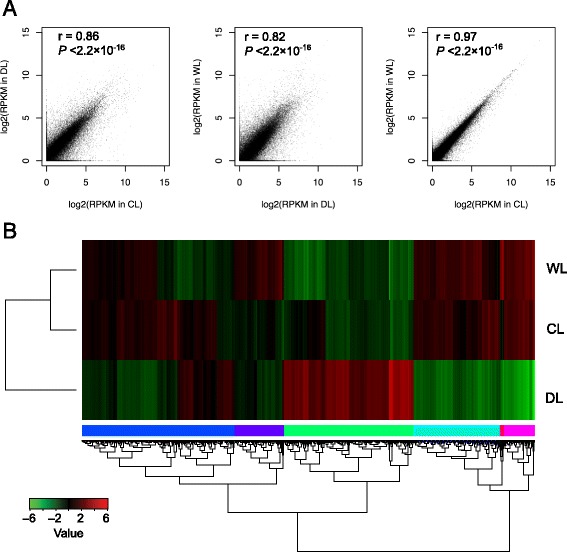
Gene expression profiles of tobacco leaves in control, drought stress, and re-watering treatment plants. Scatter plot of global gene expression for the CL vs. WL, DL vs. CL, and WL vs. DL comparisons; Pearson correlation coefficients are shown (**a**). Hierarchical clustering of all of the differentially expressed genes (DEGs) from the various comparisons (**b**)

We quantified the overall transcriptional activity of the genes in our data as reads per kilobases of exon region per million mapped reads (RPKM) and found that 49,629 protein-coding genes showed expression (>1 RPKM) in at least one sample. We further analyzed the correlation of the gene expression among the samples. The global profiles of gene expression were generally highly correlated between samples (Fig. 2a), and, as expected, the correlation of expression between CL and WL (*r* = 0.97, *P* < 2.2 × 10^−16^) was much higher than that for DL vs. CL (*r* = 0.86, *P* < 2.2 × 10^−16^) or for WL vs. DL (*r* = 0.82, *P* < 2.2 × 10^−16^). In addition, clustering analysis also indicated that the DL transcriptome was clearly distinguishable from those of CL and WL (Fig. 2b).

### Differentially expressed genes in tobacco leaves grown under drought stress

**Fig. 3:**
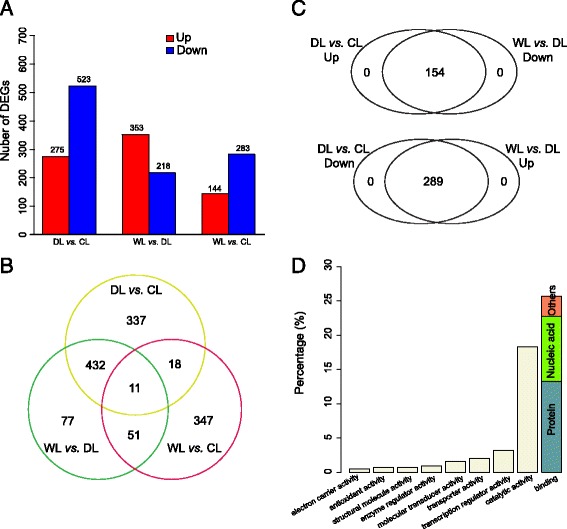
Differential gene expression analysis of control, drought stress, and re-watering treatment plants. The number of differentially expressed genes (DEGs) for the CL vs. WL, DL vs. CL, and WL vs. DL comparisons (*P* <0.05); *red*, DEGs with up-regulated expression; blue, DEGs with down-regulated expression (**a**). Venn diagram of overlapping DEGs among various comparisons (**b**). Venn diagrams to illustrate the overlap between up-regulated genes in the DL *vs*. CL comparison and the down-regulated genes in the WL *vs*. DL comparison (*upper panel*) and the overlap between the down-regulated genes in the DL *vs*. CL comparison and up-regulated genes in the WL *vs*. DL comparisons (*lower panel*) (**c**). Distribution of the various ‘Molecular Function’ GO categories of the 443 overlapping DEGs between the DL vs. CL and the WL vs. DL comparisons. The GO item “binding” can be further classified into “Protein binding” (*gray*), “Nucleic acid binding”(*green*), and “others” (*orange*) (**d**)

To identify genes that are differentially regulated under drought-stress conditions, we used DEseq software to compare the gene expression between various groupings of the three growth conditions. We identified 798, 571, and 427 DEGs between the DL and CL, WL and DL, and WL and CL comparisons, respectively (Fig. 3a and Additional files 1, 2 and 3). The overlapping DEGs among the three samples are shown as a Venn diagram in Fig. 3b. The extent of overlap of the DEGs between the WL vs. CL comparison and the other two comparisons (29 overlapping DEGs between WL vs. CL and DL vs. CL, and 62 DEGs between WL vs. CL and WL vs. DL) was much less extensive than that for the comparison between DL vs. CL and WL vs. DL (443 overlapping DEGs), suggesting that the expression of most of the drought-affected genes observed in the DL samples was returned to a ‘normal’ state following the re-watering treatment.

Detailed analysis of the 443 overlapping DEGs between the DL vs. CL and WL vs. DL comparison revealed that all of the 443 genes exhibited opposing trends in the expression patterns for the two comparisons: 154 DEGs that were up-regulated in the DL vs. CL comparison were significantly down-regulated in the WL vs. DL comparison, and 289 DEGs that were down-regulated in the DL vs. CL comparison were significantly up-regulated in WL vs. DL comparison (Fig. 3c and Additional file 4). This result strongly indicated that these 443 overlapping DEGs were highly likely to be involved in drought-stress responses in tobacco leaves. Gene ontology (GO) annotation analysis revealed that 47.2% of the 443 DEGs were classified into the “transcription regulator activity”, “catalytic activity”, and “binding” groups of the Molecular Function category (Fig. 3d). Some examples of the 443 overlapping DEGs are shown in Table 2. A set of 49 genes with significant differences in gene expression were randomly selected for validation of expression via qRT-PCR with mRNA from three individual plants (Additional files 5 and 6). The expression of 43 of these genes was highly similar in both the qRT-PCR results and the RNA-seq results.

**Table 2:** Candidate drought-responsive genes exhibiting differential expression in tobacco leaves in response to drought and re-watering treatments

Gene ID	CL (RPKM)	DL (RPKM)	WL (RPKM)	log2Fold (DL/CL)	padj (DL/CL)	Regulation (DL/CL)	log2Fold (WL/DL)	padj (WL/DL)	Regulation (WL/DL)	Annotation
Response to stimulus and stress										
gene_16580	0.7	46.91	0.37	5.81	8.59E-03	Up	−6.74	2.11E-02	Down	Late embryogenesis abundant protein
gene_64575	0.45	18.14	0	5.08	1.33E-02	Up	-Inf	6.63E-03	Down	Adenine nucleotide alpha hydrolases-like superfamily protein
gene_58070	2.55	51.67	0.63	4.10	4.92E-02	Up	−6.11	1.56E-02	Down	MLP-like protein 423
gene_71064	0	6.58	0.03	Inf	6.47E-05	Up	−7.64	1.24E-02	Down	heat shock protein 90.1
gene_24848	0.01	7.64	0.03	8.86	9.01E-05	Up	−7.87	8.14E-03	Down	heat shock protein 90.1
gene_29395	0.25	46.91	0.17	7.33	8.30E-04	Up	−7.84	8.00E-03	Down	heat shock protein 70B
gene_46118	0.32	278.97	0.55	9.52	8.52E-04	Up	−8.72	2.19E-02	Down	heat shock protein 21
gene_81903	0.02	5.45	0	8.17	8.66E-04	Up	-Inf	1.29E-02	Down	HSP20-like chaperones superfamily protein
gene_4627	0.91	49.75	0.98	5.53	4.38E-03	Up	−5.41	4.14E-02	Down	mitochondrion-localized small heat shock protein 23.6
gene_57393	0.42	14.46	0.04	4.87	1.21E-02	Up	−8.17	3.07E-03	Down	heat shock protein 101
gene_32	1.77	52.95	1.13	4.66	2.06E-02	Up	−5.29	4.52E-02	Down	mitochondrion-localized small heat shock protein 23.6
gene_26545	0.7	18.74	0.33	4.50	2.10E-02	Up	−5.56	2.82E-02	Down	heat shock transcription factor A2
gene_6132	0.59	15.69	0.32	4.50	2.19E-02	Up	−5.37	3.54E-02	Down	heat shock cognate protein 70-1
Antioxidant metabolism										
gene_31648	0	72.95	0	Inf	1.47E-05	Up	-Inf	9.23E-04	Down	glutathione S-transferase TAU 19
Cell Cycle										
gene_7843	6.75	0.03	5.81	−7.90	1.49E-04	Down	7.70	4.37E-03	Up	minichromosome maintenance (MCM2/3/5) family protein
gene_72411	6.22	0.02	4.86	−8.24	3.33E-04	Down	7.90	9.62E-03	Up	Minichromosome maintenance (MCM2/3/5) family protein
gene_47817	6.08	0.05	4.62	−7.25	6.94E-04	Down	6.87	1.73E-02	Up	Minichromosome maintenance (MCM2/3/5) family protein
gene_39576	10.34	0.16	8.29	−6.29	9.95E-04	Down	5.98	1.56E-02	Up	minichromosome maintenance (MCM2/3/5) family protein
gene_3086	9.49	0.28	7.78	−5.33	4.99E-03	Down	5.06	4.79E-02	Up	Minichromosome maintenance (MCM2/3/5) family protein
gene_50222	4.61	0	5.25	-Inf	4.36E-03	Down	Inf	1.52E-02	Up	Cyclin family protein
gene_69883	3.7	0	3.43	-Inf	4.42E-03	Down	Inf	2.72E-02	Up	Cyclin B2
gene_10225	5.91	0.13	7.51	−5.70	1.01E-02	Down	6.06	2.63E-02	Up	CYCLIN B2
gene_64406	2.63	0	3.41	-Inf	2.12E-02	Down	Inf	3.34E-02	Up	Cyclin family protein
gene_11897	3.75	0.07	5.52	−6.05	2.41E-02	Down	6.63	2.82E-02	Up	Cyclin family protein
Photosynthesis										
gene_36062	58.44	0	91.53	-Inf	1.53E-05	Down	Inf	9.23E-04	Up	chlorophyll binding (GO:0016168)
gene_36065	543.31	0.27	786.01	−11.22	1.76E-02	Down	11.51	>0.05	Up	chlorophyll binding (GO:0016168)
gene_49443	130.35	0	292.05	-Inf	2.88E-05	Down	Inf	0.008004336	Up	chlorophyll binding (GO:0016168)
gene_52444	252.59	0.32	477.85	−9.88	1.53E-03	Down	10.54	>0.05	Up	chlorophyll binding (GO:0016168)
gene_62465	1.43	54.9	2.22	5.02	8.58E-03	Up	−4.63	>0.05	Down	Chlorophyll A-B binding family protein
gene_62676	126.28	7.19	163.51	−4.38	2.83E-02	Down	4.51	>0.05	Up	chlorophyll binding (GO:0016168)
gene_8497	5.7	0	6.12	-Inf	5.25E-03	Down	Inf	2.07E-02	Up	chlorophyll binding (GO:0016168)
gene_8498	208.48	0.2	236.39	−10.27	1.39E-03	Down	10.46	2.09E-02	Up	chlorophyll binding (GO:0016168)
gene_8499	265.11	0.36	350.96	−9.78	2.78E-03	Down	10.20	4.95E-02	Up	chlorophyll binding (GO:0016168)
Protein phosphorylation										
gene_43351	8.04	0.47	7.29	−4.33	3.20E-02	Down	3.96	>0.05	Up	Protein kinase protein with adenine nucleotide alpha hydrolases-like domain
gene_50346	6.14	0.17	4.75	−5.41	7.00E-03	Down	4.80	>0.05	Up	Concanavalin A-like lectin protein kinase family protein
gene_6225	0.65	24.87	0.21	5.02	1.50E-02	Up	−6.64	1.29E-02	Down	protein phosphorylation (GO:0006468)
gene_62579	12.18	0.12	17.21	−6.85	5.07E-04	Down	7.37	4.45E-03	Up	Protein kinase superfamily protein
gene_63746	16.12	0.59	20.63	−5.02	8.73E-03	Down	5.39	3.64E-02	Up	Protein kinase superfamily protein
gene_65325	4.58	0.12	2.71	−5.51	4.49E-03	Down	4.50	>0.05	Up	Concanavalin A-like lectin protein kinase family protein
gene_73172	1	0.01	0.86	−6.53	4.56E-02	Down	6.43	>0.05	Up	Protein kinase family protein with ARM repeat domain
gene_81940	5.89	0.05	6.12	−7.22	7.22E-03	Down	7.28	3.29E-02	Up	cyclin-dependent kinase B1
gene_45400	0.16	3.36	0	4.39	>0.05	Up	-Inf	1.89E-02	Down	protein phosphorylation (GO:0006468)
gene_51323	1.76	0	3.51	-Inf	>0.05	Down	Inf	4.91E-02	Up	Serine_threonine-protein kinase 6

### Functional classification of differentially expressed genes

**Fig. 4:**
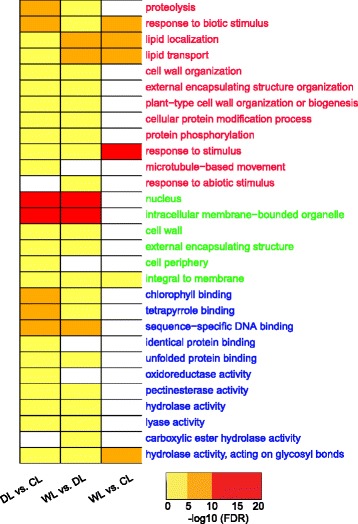
Heat map showing the differential enrichment of GO terms among the CL vs. WL, DL vs. CL, and WL vs. DL comparisons. A FDR cutoff of 0.01 was used to select the enriched GO terms. The text with font color in *red*, *green*, and *blue* indicate, respectively, the GO terms in the biological processes, cellular components, and molecular function catagories

To better understand the function of the DEGs that we detected among the sample comparisons, we first conducted GO enrichment analysis on the significantly up- and down-regulated genes that were detected by pair-wise comparisons in the CL, DL, and WL using AmiGO [30]. In total, we identified 29 significant GO categories. We found that 24 categories were only significantly enriched with the DEGs the from DL *vs*. CL comparison or the WL *vs*. DL comparison, which might be associated with drought-stress responses in tobacco leaves (Fig. 4). For biological processes, DEGs related to “cell wall organization”, “protein phosphorylation”, and “response to abiotic stimulus” were enriched in the DL *vs*. CL comparison or the WL *vs*. DL comparisons. For molecular functions, 11 GO categories including “tetrapyrrole binding”, “sequence-specific DNA binding”, “oxidoreductase activity”, and “pectinesterase activity” were enriched among the DEGs.

An informative analysis of the functional annotations among a set of DEGs can be achieved by studying the enrichment of DEGs in a particular pathway. KEGG (Kyoto Encyclopedia of Genes and Genomes) pathways analysis revealed that 10 pathways including “Cell Cycle”, “DNA replication”, “Fatty acid elongation”, “Sesquiterpenoid and triterpenoid biosynthesis”, and “Photosynthesis-antenna proteins” were enriched with DEGs in tobacco leaves grown under drought stress; these were detected in only in DL *vs.* CL comparison or the WL *vs.* DL comparison (Table 3).

**Table 3:** KEGG pathway enrichment analysis of differently expressed genes in the three tobacco leaf sample libraries

Comparison	Pathway ID	Pathway Name	Q value	Fold Enrichment
DL *vs* CL	ko03030	DNA replication	8.55E-07	6.56
	ko04110	Cell cycle	8.55E-07	4.36
	ko00196	Photosynthesis - antenna proteins	1.69E-06	8.55
	ko00909	Sesquiterpenoid and triterpenoid biosynthesis	3.51E-05	12.36
	ko00040	Pentose and glucuronate interconversions	1.38E-04	4.24
	ko04914	Progesterone-mediated oocyte maturation	5.84E-04	4.70
	ko04115	p53 signaling pathway	1.05E-03	4.80
	ko00062	Fatty acid elongation	3.18E-03	6.33
	ko00592	alpha-Linolenic acid metabolism	6.01E-03	4.54
	ko00591	Linoleic acid metabolism	1.04E-02	4.84
	ko00966	Glucosinolate biosynthesis	2.94E-02	10.33
	ko00909	Sesquiterpenoid and triterpenoid biosynthesis	2.18E-04	13.56
	ko00040	Pentose and glucuronate interconversions	2.75E-04	4.77
	ko04110	Cell cycle	4.95E-04	3.65
	ko03030	DNA replication	5.46E-04	5.25
	ko00196	Photosynthesis - antenna proteins	8.85E-04	6.52
	ko04914	Progesterone-mediated oocyte maturation	1.75E-03	4.84
	ko04115	p53 signaling pathway	4.48E-03	4.71
	ko00966	Glucosinolate biosynthesis	1.32E-02	14.17
	ko00592	alpha-Linolenic acid metabolism	4.21E-02	3.74
WL *vs* CL	ko00591	Linoleic acid metabolism	4.23E-03	9.49
	ko00590	Arachidonic acid metabolism	4.72E-03	8.34
	ko04146	Peroxisome	6.77E-03	4.55
	ko00140	Steroid hormone biosynthesis	1.10E-02	6.13
	ko04626	Plant-pathogen interaction	1.69E-02	3.57
	ko00710	Carbon fixation in photosynthetic organisms	3.49E-02	3.51

### Transcription factor analysis

Transcription factors are widely involved in various biological processes and play important roles in plant responses to abiotic stress. In tobacco, 5603 TF-encoding genes were found and classified into 80 different families by sequence alignment against the Plant Transcriptional Factor Database [31]. Based on our sequence analyses, a total of 3465, 3361, and 3378 TF-encoding genes were detected in CL, DL, and WL, respectively.

**Fig. 5:**
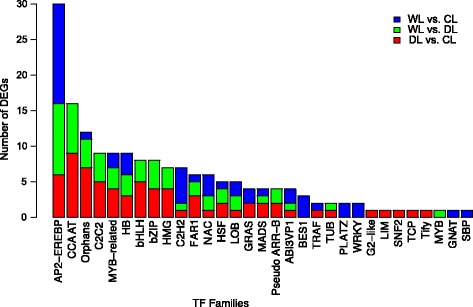
Distribution of differentially expressed transcription factors in the DL vs. CL, WL vs. DL, and WL vs. CL comparisons

Further analyses revealed that 69 TF-coding genes of 25 TF families were differentially expressed between DL and CL, and among these the TF gene family with the highest number of DEGs was the CCAAT family (CCAAT-box binding CBF; 13.04%) followed by Orphans (10.14%), AP2-EREBP (or AP2/ERF; 8.69%), bHLH (7.24%), bZIP (5.79%), and MYB-related (5.79%) families (Fig. 5 and Additional file 1). Similarly, in the comparison between WL and DL, the identified DEGs included a total of 56 TF-encoding genes from 19 TF families: and most of them also belong to AP2-EREBP (17.86%), CCAAT (12.50%), Orphans (7.14), bZIP (7.14), bHLH (7.14%), and MYB-related (5.35%) families (Additional file 2). As for TFs of interest to drought-stress responses, we found that genes in the CCAT, C2C2, bZIP, bHLH, and HMG families were specifically induced under drought conditions.

### Deep sequencing results of small RNA Libraries

To investigate the miRNA component of small RNAs and the dynamic changes of the miRNAs under the drought-stress conditions, we constructed small RNA libraries using total RNA and a total of 30,374,218, 24,325,393, and 23,388,818 filtered high-quality reads that were obtained from the CL, DL, and WL, respectively. The size distribution of reads was not significantly different in the three libraries, and the majority of small RNAs were in the range from 18 to 24, with 24 nt as the most frequent size (Additional file 7). These results are consistent with previous studies in tobacco [32, 33] and similar to results reported for *M. truncatula* [34], maize [35], potato [36], tomato [37], *Citrus trifoliate* [38], *Citrus sativus* [39], *Arabidopsis* [40], and rice [41]. In order to remove rRNA, tRNA, snRNA, and snoRNA, all clean reads of three libraries were analyzed by BLAST against the Rfam database (See Methods section for details; these small RNAs accounted for 158,145, 197,174, and 1,943,313 total reads in CL, DL, and WL, respectively (Additional file 8).

### Detection of known and novel miRNAs in tobacco leaves

The investigation of both known miRNA and novel putative miRNAs were conducted by miRDeep2 program [42]. This program combined the position and frequency of small RNAs with the secondary structure of miRNA precursors to provide novel miRNAs which may specifically find in tobacco. In total we discovered 63 known tobacco miRNAs (49, 44, and 50 in CL, DL, and WL, respectively) and 20 (35.7%) of the known miRNAs were detected in all three libraries, while 40 (71.4%) were shared in at least two of three libraries (Table 1 and Additional file 9). Further analysis revealed that the 63 known miRNAs belonged to 32 miRNA families, and miR166 and miR6149 were the most extensively-represented families, totally accounting for 76.8% ~ 88.4% of the small RNA reads supporting the known tobacco miRNAs in three libraries (Additional file 10).

We also found 368 novel miRNA candidates in the three libraries (Additional files 11, 12 and 13). The secondary structures of typical stem-loop hairpins in novel pre-miRNAs with their alignments with sequenced small RNAs helped to identify their precursors. Among the novel miRNAs, 53 (14.4%) were detected in all three libraries, with NovelmiRNA-327, NovelmiRNA-254, and NovelmiRNA-150, as the most abundant miRNAs (Additional file 14).

### Differential expression analysis of miRNAs and target prediction of miRNAs

**Fig. 6:**
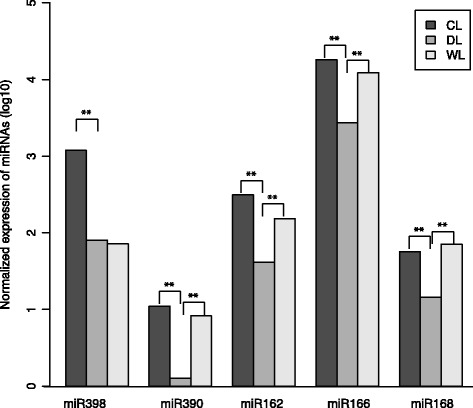
The expression profiles of the normalized sequence reads of the known miRNA families in the drought stress library (DL) relative to those of the untreated-control library (CL) and re-watering library (WL). ** *P*-values < 0.01 are considered to indicate highly significant differences

We compared the expression of miRNAs in three libraries based on a Poisson distribution approach [43]. For the known miRNA in tobacco, we identified those five miRNA families, including miR398, miR390, miR162, miR166, and miR168, were differential expressed among DL and CL or WL libraries (Fig. 6). Similarly, 32 differentially expressed novel miRNAs were found between CL and DL, and 44 were found between WL and DL. miR398, miR390, miR162, miR166, and miR168 were selected for validation via qRT-PCR analysis (Additional files 15 and 16). We used the miRanda [44] program to explore the biological significance of miRNAs and predict the biological targets of the DEMs. The miRNA negatively regulates target mRNA in their translational repression or mRNA degradation. Analysis to identify negative correlations between the expression of DEMs and DEGs revealed 92 potential mRNA-miRNA interactions between CL and DL and 32 potential mRNA-miRNA interactions between WL and DL (Additional file 17). Considering that some mRNAs were putatively targeted by multiple miRNAs, we identified a total of 64 DEGs that were putatively targeted by 25 DEMs. GO annotation analysis revealed that 40.5% and 47.6% of the DEGs were classified into “catalytic activity” and “binding” for the Molecular Function category (Additional file 18).

## Discussion

### mRNA profiling in tobacco leaves under stress

To achieve a goal in this study of investigating a wide range of drought-response genes in tobacco to dissect the physiological, metabolic, and cellular process through drought-stress, we conducted the experiments of high-throughput sequencing technology to find out the transcriptome changes in the leaves of tobacco undergoing drought-stress and re-watering treatment. The transcripts of 49,629 genes were detected, accounted for expression of 60% predicted genes in tobacco genome, while about 14.6% of the reads could not be matched to any genes in three libraries; these likely represent unidentified genes, genes expressed in tissues that we did not sample, or genes from incompletely-sequenced regions in tobacco genome. Our experimental design with the re-watering treatment allowed for multi-way comparisons of differential expression analysis, and the trends for the DEGs among the CL vs. DL and the WL vs. DL comparisons were highly similar, strongly suggesting that the 443 DEGs we detected are in some way involved in drought-stress responses in tobacco leaves.

As would be expected, the GO terms ‘response to stimulus’ and ‘response to abiotic stimulus’ were highly enriched with the DEGs (12 genes), highlighting the efficacy of the drought treatments in these experiments for eliciting relevant responses and the seeming reliability of the gene expression data (Fig. 4). The gene_with the identifier 16580, annotated as a late embryogenesis abundant protein, was significantly up-regulated (5.8 fold) under drought stress, while its expression decreased (6.7 fold) after re-watering treatment (Table 2). Late embryogenesis abundant (LEA) proteins are proteins in both plants and animals that protect other proteins from aggregation due to drought stress or osmotic stresses associated with low temperature [45]. Heat-shock proteins (HSPs) are usually induced to help plants cope with heat and other environmental stresses. Such as, *Trichoderma harzianum* HSP70 is involves in abiotic stress in *Arabidopsis* [46]. Our study strongly suggests the potential roles of LEA proteins and HSPs in response to drought stress in tobacco.

To perform the abiotic stress tolerance, antioxidant defense systems composed of antioxidant enzymes and non-enzymatic antioxidants scavenging the reactive oxygen species such as superoxide radicals, hydrogen peroxide and hydrogen peroxide (H_2_O_2_) and hydroxyl radicals in cells experiencing abiotic stresses [47]. In our study, we found 7 DEGs annotated as antioxidant enzymes in the DL vs. CL and WL vs. DL comparisons, including gene_6938 (POD superfamily), gene_6846 (POD superfamily), gene_9311 (POD superfamily), gene_24447 (POD superfamily), gene_62798 (POD superfamily), gene_76922 (POD superfamily), and gene_31648 (GST). Previous studies showed that genes encoding Glutathione S-transferases were significantly up-regulated under drought stress in rice [14], maize [16], *C. colocynthis* [18], Pinaceae [48] and tobacco [28]. Consistent with these reports, our study revealed that gene_31648, annotated as Glutathione S-transferase, was the most significantly different DEGs between DL and CL; it was significantly up-regulated in tobacco leaves under drought stress compared to both the control and the re-watering treatment, with RPKM values of 0, 72.95, and 0 in the CL, DL, and WL samples, respectively (Table 2).

Transcription factors play essential roles in diverse biotic/abiotic stresses by activating expression of downstream regulatory and structural genes that enact plant stress responses [49]. TFs are known to be involved in ABA signal transduction in both the ABA-dependent (MYB, MYC, and NAC) and ABA-independent (AP2/ERF) stress response pathways. In our study, the TF families with the most abundantly expressed (number of) of DEGs in tobacco leaves under drought stress were the AP2-EREBP (AP2/ERF), CCAAT (CBF), MYB-related, bHLH, and bZIP families (Fig. 5). All of these TFs have been previously reported to be involved in the transcriptional regulation of abiotic stress tolerance in plants [49, 50]. Thus, the differential expression of these TFs in our study seems highly reasonable and strongly bolsters the conclusion that these are likely to be functional genes in tobacco responses to drought stress. We found 18 DEGs annotated as being involved in the cell cycle regulatory pathway; these showed decreased expression levels in leaves under drought stress as compared with the control, and of these 11 DEGs were significantly up-regulated following the re-watering treatment (Table 2). Cell cycle is synergistically regulated by the co-operation of many cellular participants and synchronized events. The cells would die when the cell cycle was blocked due to insufficient cellular resources or other stress conditions [51]. In response to the drought stress in tobacco leaves, a host of genes encoding cell cycle factors showed decreased transcripts, suggesting that tobacco leaves under drought stress appears to experience a dramatic perturbation of the cell cycle, which likely prevents the further proliferation of such cells.

As a complex metabolic process, photosynthesis is well studied and is known to be highly responsive to drought stress. Drought stress is typically accompanied by stomatal closure, reduced mesophyll CO_2_ diffusion, and decreased rates of photosynthesis [52]. The down regulation of photosynthesis-related genes in response to drought stress has been reported for various plants, including as rice [53], *C. colocynthis* [18], soybean [15], and *Ammopiptanthus mongolicus* [54]. Our pathway enrichment analysis suggested that nine DEGs were associated with the KEGG term “Photosynthesis - antenna proteins” (Table 3). Similarly, GO analysis indicated that that eight genes were classified into the “chlorophyll binding” GO term (Fig. 4). All ten of the photosynthesis-related DEGs in our study, with one exception, were drastically down-regulated in tobacco leaves under drought-stress. Only gene_62465, which is annotated as a chlorophyll a-b binding family protein, had increased expression under drought stress. Studies in *A. thaliana* have indicated that photosynthetic responses to drought stress are highly complex and involve the alteration of the expression patterns of a multitude of genes [55]. Our observations that many photosynthesis-related genes were among the DEGs associated with drought stress, in combination with the results for the antioxidant-responsive DEGs, highlight the complexity of the relationships between photosynthesis and drought stresses (both oxidative and osmotic) in plants.

### miRNA profiling in tobacco leaves under stress

Many recent studies have demonstrated that plant miRNA molecules are involved in cellular responses to abiotic stress. This has been demonstrated with salt, cold, and drought stress experiments [56–58]. Here, we performed small RNA sequencing on the CL, DL, and WL samples and identified 63 previously-known miRNAs from 32 families known and identified 368 novel candidate miRNAs. Many miRNAs with a wide range of expression levels were found in the CL, DL, and WL libraries. Consistent with a previous miRNA study in tobacco [29], the most abundantly expressed miRNA family across the three libraries was the miR166 family. This family includes miR166a, miR166b, miR166c, miR166d, miR166f, miR166g, and miR166h (Additional files 9 and 10).

We found significant down regulation of miR398, miR390, miR162, miR166, and miR168 under drought stress (Fig. 6). Integrated analysis of the miRNA and mRNA levels revealed 92 mRNA-miRNA interactions (i.e., negative correlations) between the CL and DL plants and 32 mRNA-miRNA interactions between the DL and WL plants. MiR398 has been proposed to participate in the plant regulatory networks in responses to oxidative stress, water deficit, salt stress, ultraviolet stress, copper and phosphate deficiency, high sucrose levels, bacterial infection, and ABA signalling [59]. Previous studies have shown that down regulation of miR398 in response to drought stress facilitates the up-regulation of CSD2 (copper/zinc superoxide dismutase), and thereby helps plants cope with oxidative stress [60]. In our study, we found that miR398 was down-regulated under drought stress, which would be expected to lead to increase in antioxidant (e.g. SOD) activity. The drought-induced down regulation of miR398 in tobacco is consistent with the results reported maize [61] but it is contrast to the results reported by Trindale et al. for *M. truncatula* [62] and wild emmer wheat [57]. Also of note, and consistent with a previous study of miRNA expression in tobacco [28], miR162 had decreased expression in the DL samples as compared with the CL (7.5 fold down regulation) and WL (3.7 fold down regulation) samples. Moreover, we observed the down regulation miR168 expression in response to drought; this miRNA molecule has been shown to be drought responsive in *Arabidopsis* [63].

Our combination of data for mRNA and small RNA sequencing from tobacco leaf samples from plants grown under control, drought, and re-watering treatment enabled us to perform a combined analysis of the drought-stress responsive genes and miRNAs in tobacco. We identified many DEGs that are potentially involved in drought-stress responses, including genes related to responses to stimulus and stress, antioxidant defense systems, the cell cycle, and photosynthesis. We were also able to use our miRNA data in combination with our mRNA data to identify many putative mRNA-miRNA interactions that may plant important, higher-order molecular genetic regulator roles (i.e., post transcriptional) in the drought-stress responses of tobacco plants. These findings provide valuable information about potentially novel miRNA families that have regulatory functions and establish an empirical foundation that will facilitate further functional characterization studies of the genes and miRNAs involved in drought-stress responses in tobacco.

## Conclusion

In present studies, we generated mRNA and small RNA sequencing of tobacco leaves under drought and re-watering treatment and performed comprehensive analysis of the drought-responsive genes and miRNAs. The results revealed the DEGs that potentially involved in drought stress response, including genes related to response to stimulus and stress, antioxidant defense system, cell cycle, photosynthesis process, cell wall adjustments and protein phosphorylation. In addition, our analysis also identified miRNAs that may plant important roles in tobacco leaves responding to drought stress. These findings provide valuable information for further functional characterization of genes and miRNAs in response to abiotic stress in general and drought stress in tobacco.

## Methods

### Plant materials and growth conditions

Tobacco line K326 was used in the present study. The K326 originated from a cross of two breeding lines which obtained from the cross breeding of Coker 139, Coker 319 and McNair 30, NC 95, respectively, and released in 1982 by Novartis Seeds, Inc.. The variety is susceptible to tobacco mosaic virus, low resistant to black shank disease and Granville wilt, while resistant to root-knot nematodes. The particular K326 seeds that were used in this study were purchased from the Tobacco Research Institute of the Chinese Academy of Agricultural Sciences in Qingdao, China, and were germinated in MS medium. Two weeks after germination, the seedlings were transferred to pots (Metro-Mix 200; Sun Gro, USA). After three weeks of growth in the pots in a greenhouse, tobacco plants at highly similar stages of growth were selected and divided into three groups. The treatment period lasted for 10 days (sample collection on 10 day). CL plants were watered with 1000 mL water on days 1, 4, and 7. DL plants received no water for ten days. WL plants were re-watered, to full soil media saturation, on day 7. Leaves from ten plants were collected for each group. These materials were divided into two parts and frozen prior to analysis. One portion of each of ten samples for each group was combined together as a single, pooled sample for the RNA extractions used in the RNA-seq analysis. For the water content and chlorophyll fluorescence measurements, seven plants from each group were evaluated.

### Leaf water content

The third to the sixth leaves from the tops of tobacco plants were removed and rinsed with distilled water. After blotting to remove excess water, the fresh weights of these leaves were determined. The dry weights were determined after the leaves were dried at 70 °C for 48 h. According to the formula of WC = 100% ╳ (FW-DW)/FW, the water content of the leaves (WC, %) was calculated [64]. Seven individual plants were used for replicates in all measurements.

### Chlorophyll fluorescence test

The fourth leaves from the top of plants were measured for chlorophyll fluorescence according to the description by Zhou and Qiu [64]. Briefly, all plants were dark-adapted for 15 min before measurement. Then, the maximum chlorophyll fluorescence (Fm) and variable fluorescence (Fv) of attached leaves was detected with a Plant Efficiency Analyzer (Handy PEA; Hansatech Instruments Ltd, UK) using a leaf clip. The actinic light (3600 μmol m^−2^s^−1^) was provided by an array of three high-intensity light-emitting diodes [64]. All measurements were replicated with leaves from seven individual plants.

### Construction and sequencing of mRNA-Seq and small RNA libraries

For information about sample pooling, see the sub-section about the plant materials, above. Total RNA was extracted from leaves using Trizol reagent (Invitrogen) and RNA integrity was assessed by an Agilent BioAnalyzer 2100. Equal amounts of RNA from CL, DL, and WL samples were used for construction of mRNA-Seq and small RNA libraries. The mRNA-Seq libraries were prepared using an Illumina TruSeq RNA Sample PreKit following the manufacturer’s protocols. Briefly, mRNA was purified from total RNA using poly-T oligo-attached magnetic beads, fragmented, and reverse transcribed into cDNA. Adapters were then ligated on to the cDNA molecules and the fragments were amplified by PCR. The sequencing was performed in paired-end reads (2 × 101 bp) using the Illumina Hiseq 2500 sequencing platform. For the small RNA sequencing, RNA bands of around 18–30 nt in length were isolated. Libraries were prepared according to the Small RNA Sample Preparation Protocol (Illumina) and were sequenced with the Illumina Hiseq 2500 sequencing platform with 50 bp single-end reads. The quality of data from mRNA and small RNA sequencing was evaluated by the FastQC method (http://www.bioinformatics.bbsrc.ac.uk/projects/fastqc/), and all of the raw data was deposited in the SRA database (http://www.ncbi.nlm.nih.gov/sra/) with accession number SRP071695.

### Processing and mapping of mRNA-Seq reads

The Illumina Hiseq platform generated the mRNA-Seq reads which removed the adaptor sequences and the low-quality (<20) bases at the 5′ and 3′ ends by Trimmomatic (v0.30) [65], and reads longer than 70 bp were used for further experiment. The reads were mapped to the tobacco genome (*Nicotiana tabacum* from ftp://ftp.solgenomics.net/genomes/Nicotiana_tabacum/) using bowtie2 (2.1.0) [66] with default parameters after preprocessing of mRNA-Seq data. Gene expression levels were presented as FPKM (fragments per kilo bases per million reads) values [67]. Genes with expression levels >1 FPKM were retained for further analysis.

### Detection of differentially expressed genes

We used sequence counts corresponding to annotated genes as inputs identification of differentially expressed genes with DEseq software [68] (www.bioconductor.org). This tool uses a negative binomial distribution model to test for differential gene expression. We adjusted for multiple testing using FDR correction and a very stringent cutoff, FDR < 0.05, and more than a 2 fold change, as the criteria to classify ‘differentially expressed’ genes between two-way pairing among the three experimental conditions (i.e., CL vs DL; DL vs. WL; CL vs. WL).

### Gene ontology and KEGG pathway enrichment analysis

AmiGO with the default parameters were used to obtain gene ontology terms of each gene and analyze GO functional enrichment by using hypergeometric tests with FDR correction to obtain an adjusted *P*-value between particular test gene groups and the whole annotation data set, respectively. The differentially expressed genes in KEGG pathway was analyzed using Cytoscape [69] with the ClueGO plugin [70].

### Transcription factor analysis

The transcription factors in tobacco were predicted by aligning the gene sequences against the Plant Transcriptional Factor Database (http://planttfdb.cbi.pku.edu.cn/) [31] with BLAST (evalue = 1e-5); the tobacco genes were classified according to their TF families.

### Processing and mapping of small RNA sequencing data

Clean reads were screened from raw sequencing reads by removing adaptors, poly A sequences, and low-quality bases at both ends of small RNA reads. Sequences shorter than 18 nt or longer than 32 nt, after trimming, were removed. The high-quality clean reads were mapped to the tobacco genome using bowtie2 (2.1.0) [66]. To remove small RNAs originating from rRNA, tRNA, snRNA, and snoRNA, we also mapped the short reads to the Rfam database (http://rfam.xfam.org/, version11) with BLASTN (word_size =10, evalue = 1e-5). The reads that mapped onto Rfam were discarded.

### Identification of known and novel miRNAs, and prediction of the targets of miRNAs

The un-annotated small RNA reads that remained after the elimination of other non-coding RNAs were mapped to the reference tobacco genome; the reads with multi-hits against genome were filtered out. The remaining potential miRNA reads were analyzed using the miRDeep2 [42] pipeline to identify both known and putative microRNAs in tobacco. The miRNA targets were predicted using miRanda [44] with a score ≥155 and predicted energy ≤ −20 kcal/mol.

### Detection of differentially expressed miRNA molecules

Evaluation of the inferential statistical significance of differences in the comparison of miRNA expression between two particular libraries was based on a previously established model [43]. We adjusted for multiple testing using FDR correction and a very stringent cutoff, FDR < 0.05, and more than a 2 fold change, as the criteria to classify ‘differentially expressed’ genes between two-way pairing among the three experimental conditions.

### qRT-PCR analysis to verify the RNA-seq results for mRNA and miRNA expression

Leaves from three plants of each group were used for conductance of qRT-PCR. Total RNA was extracted from leaf tissue of the previously collected and frozen sample portions from the original drought experiment. The cDNA was generated using PrimeScriptTM One Step RT-PCR Kit Ver. 2 (Takara, RR055A) and diluted 10 times as the templates for qPCR. For the miRNA expression analysis, the stem-loop pulsed reverse transcription was performed to generate cDNA [71], which was diluted five times as the template for qRT-PCR. qRT-PCR reactions were performed using 2 × SYBR Green qPCR Master Mix (Biotool, B21206) on an ABI StepOneTM instrument. Three independent biological replicates and three technical replicates of each biological replicate were conducted for qRT-PCR analyses. The tobacco *actin* gene was used as the reference gene for data normalization in the mRNA analysis, and a tobacco U6 sequence was used as the reference for data normalization in the miRNA analysis. The relative expression (as a fold change) of each sample was calculated using the 2-ΔΔCT method [72]. Primers used in these qRT-PCR analyses are shown in Additional files 6 and 16.

## Additional files

Additional file 1: List of differentially expressed genes between DL and CL (DL vs. CL) in tobacco leaves. (XLSX 151 kb)
Additional file 2: List of differentially expressed genes between WL and DL (WL vs. DL) in tobacco leaves. (XLSX 118 kb)
Additional file 3: List of differentially expressed genes between WL and CL (WL vs. CL) in tobacco leaves. (XLSX 103 kb)
Additional file 4: The 443 overlapping differentially expressed genes between DL vs. CL and WL vs. DL. (XLSX 105 kb)
Additional file 5: Relative gene expression analysis of significant differences assessed via qRT-PCR. (XLSX 14 kb)
Additional file 6: Primer sequences used in qRT-PCR analysis for mRNA validation. (XLSX 15 kb)
Additional file 7: Distribution of small RNA sizes in the three libraries. (PDF 4 kb)
Additional file 8: Summary of the number of reads for other non-coding RNAs in three the libraries. (XLSX 34 kb)
Additional file 9: Summary information of the known tobacco miRNAs and their abundance as identified in the three libraries. The expression of miRNA was normalized as transcripts per million reads (TPM) values. (XLSX 54 kb)
Additional file 10: Summary information of miRNA families and their abundance identified in the three libraries. The expression of miRNA was normalized as transcripts per million reads (TPM). (XLSX 53 kb)
Additional file 11: Novel miRNA candidates detected in th eCL library. (XLSX 27 kb)
Additional file 12: Novel miRNA candidates detected in the DL library. (XLSX 64 kb)
Additional file 13: Novel miRNA candidates detected in the WL library. (XLSX 67 kb)
Additional file 14: Summary information of the novel miRNAs and their expression levels in the three libraries. The expression of miRNA was normalized as transcripts per million reads (TPM). (XLSX 73 kb)
Additional file 15: qRT-PCR relative gene expression analysis of miR398, miR390, miR162, miR166, and miR168. (PDF 340 kb)
Additional file 16: Primer sequences used for the qRT-PCR validation of miRNA expression. (XLSX 13 kb)
Additional file 17: Instances of negative correlation from comparisons of the expression patterns of differentially expressed genes and differentially expressed miRNAs in the DL vs. CL and WL vs. DL. comparisons. (XLSX 52 kb)
Additional file 18: GO annotation of differentially expressed genes targeted by differentially expressed miRNAs. (JPG 201 kb)

## Abbreviations

ABA: Abscisic acid
APX: Ascorbate peroxidase
CAT: Catalase
DEGs: Differentially expressed genes
DEM: Differentially expressed miRNA
GO: Gene ontology
GPX: Guaiacol-peroxidase
GST: Glutathione S-transferase
HSPs: Heat-shock proteins
KEGG: Kyoto Encyclopedia of Genes and Genomes
LEA: Late embryogenesis abundant
MCMs: Minichromosome maintenances
POD: Peroxidase
RPKM: Reads per kilobases of exon region per million mapped reads
SOD: Superoxide dismutases
TF: Transcription factor
