# Supplementary figures and images for: Integrated mRNA and microRNA analysis identifies genes and small miRNA molecules associated with transcriptional and post-transcriptional-level responses to both drought stress and re-watering treatment in tobacco
Source: BMC Genomics. 2017 Jan 10;18:62. doi: 10.1186/s12864-016-3372-0 (PMC5223433; doi:10.1186/s12864-016-3372-0)

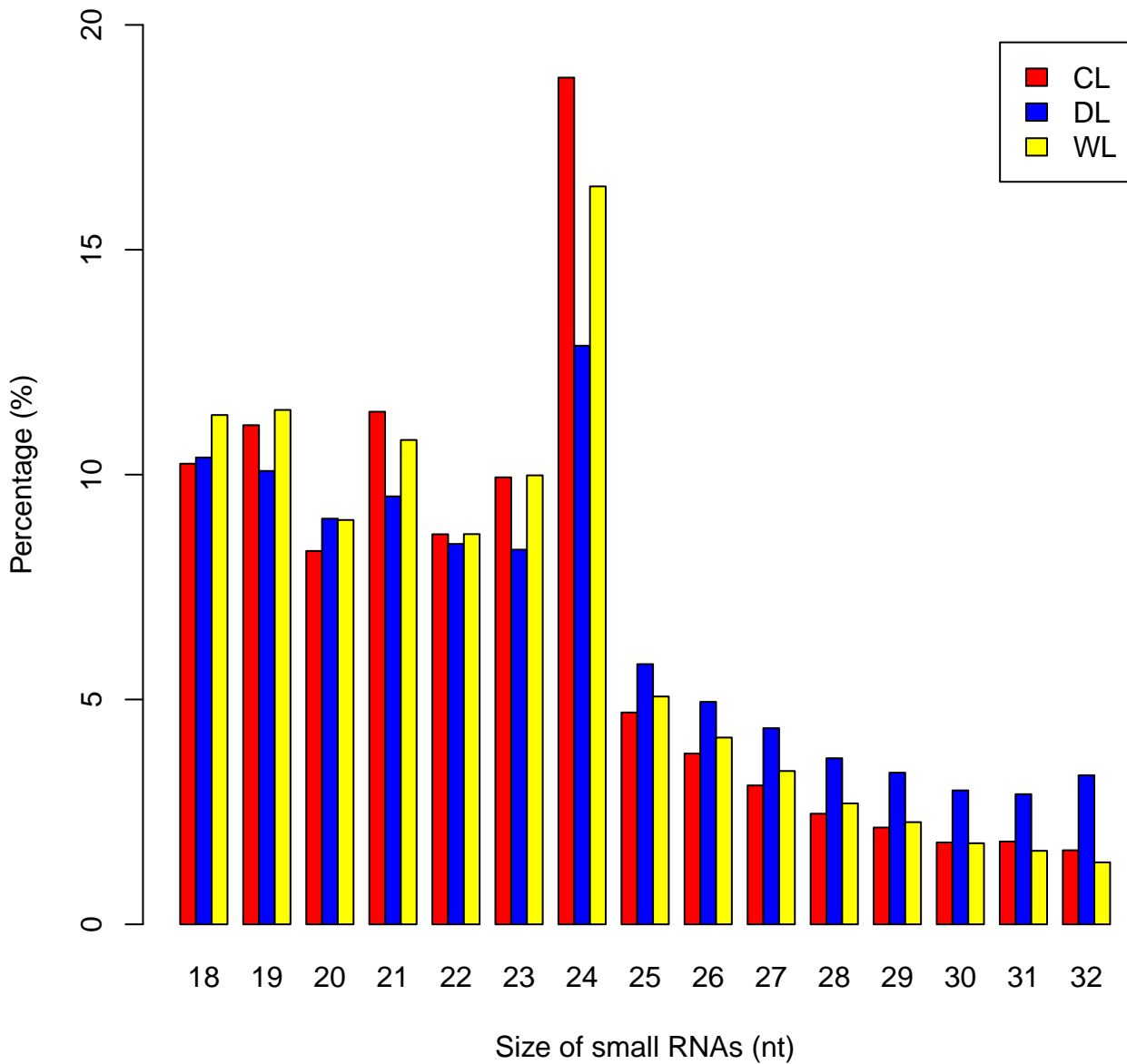

Supplement: Additional file 7: — Distribution of small RNA sizes in the three libraries. (PDF 4 kb) [file 12864_2016_3372_MOESM7_ESM.pdf]

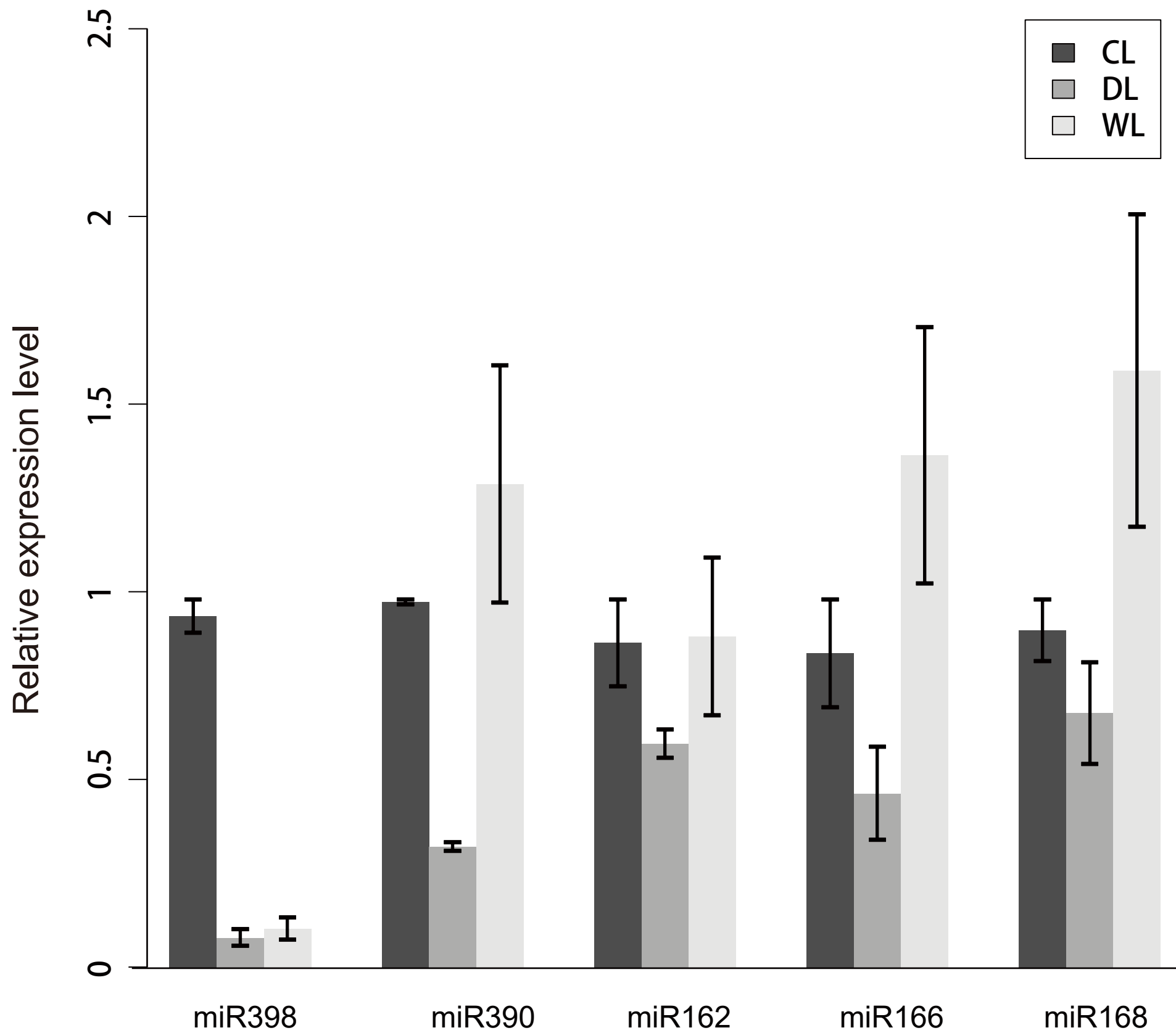

Supplement: Additional file 15: — qRT-PCR relative gene expression analysis of miR398, miR390, miR162, miR166, and miR168. (PDF 340 kb) [file 12864_2016_3372_MOESM15_ESM.pdf]

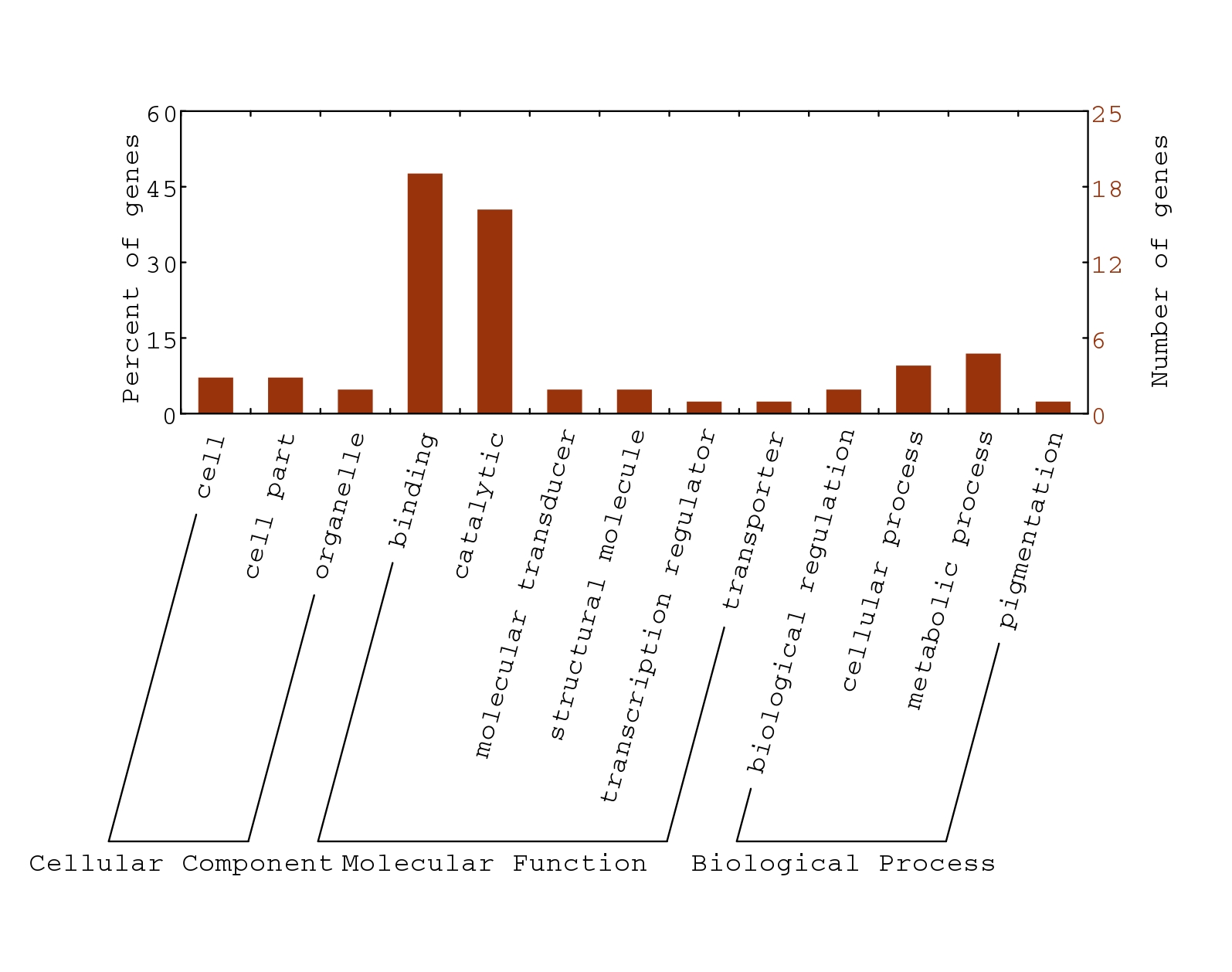

Supplement: Additional file 18: — GO annotation of differentially expressed genes targeted by differentially expressed miRNAs. (JPG 201 kb) [file 12864_2016_3372_MOESM18_ESM.jpg]
